# Deconstructing Markush: Improving the R&D Efficiency Using Library Selection in Early Drug Discovery

**DOI:** 10.3390/ph15091159

**Published:** 2022-09-18

**Authors:** Leticia Manen-Freixa, José I. Borrell, Jordi Teixidó, Roger Estrada-Tejedor

**Affiliations:** Molecular Design Laboratory, Grup de Química Farmacèutica, IQS School of Engineering, Universitat Ramon Llull, Via Augusta 390, E-08017 Barcelona, Spain

**Keywords:** chemical space, Markush, combinatorial library, drug discovery, rational selection, drug-like molecules

## Abstract

Most of the product patents claim a large number of compounds based on a Markush structure. However, the identification and optimization of new principal active ingredients is frequently driven by a simple Free Wilson approach, leading to a highly focused study only involving the chemical space nearby a hit compound. This fact raises the question: do the tested compounds described in patents really reflect the full molecular diversity described in the Markush structure? In this study, we contrast the performance of rational selection to conventional approaches in seven real-case patents, assessing their ability to describe the patent’s chemical space. Results demonstrate that the integration of computer-aided library selection methods in the early stages of the drug discovery process would boost the identification of new potential hits across the chemical space.

## 1. Introduction

The pharmaceutical industry has always been challenged in improving the research and development (R&D) efficiency [1,2]. Following the *fail early* strategy, having a wide range of molecular structures in the initial stages is crucial to increase the chances of finding a compound with biological activity against the target under study. In this sense, the exploration of molecular diversity during the hit or lead discovery phases plays a pivotal role. The description and exploration of the drug-like chemical space (the so-called drugspace [3]) has always been of great concern given the overwhelming number of molecules that can be obtained by fragment combination (which was barely estimated to be 10^60^ small molecules) [4]. In light of this scenario, finding new molecules with optimal drug-like properties becomes as difficult as finding a needle in a haystack, at least without the support of computer-aided techniques.

Many cheminformatic tools have been developed for better mapping of the chemical space by defining each molecule in a chemical library as a point in the molecular descriptor’s space (or using principal component analysis): distance-based approaches [5,6,7,8], cluster-based selections [9], cell-based selections [10,11] or optimization-based selections [12,13]. These techniques allow the rational navigation through the chemical space (a process known as chemography [3,14,15,16,17]), contributing to hit discovery [18,19,20,21] and the identification of unexplored regions that might hide biologically active compounds. Previously reported results in our group [22,23] proved that rational selection methods did improve the hit identification step when studying a library containing 125,396 analogs of HEPT, an inhibitor of HIV-1 reverse transcriptase. The goodness of 11 available diversity selection methods was assessed and the final selection of 25 compounds that covered 90% of the chemical space showed a broad activity range (with EC_50_ between 0.05 μM and >90 μM), even better than the reference compound (HEPT, EC_50_ = 3.3 μM).

Markush structures stated in the claims part of a product patent delimit the chemical space covered by a patent. With the appearance of a combinatorial library, they describe the analogs of a certain drug by defining a molecular skeleton that contains one or more variable substructures listed and represented by particular or generic fragments [24,25]. It is worth noting that the description of a Markush structure does not imply the proven synthesis of all the compounds derived from the combinatorial library. In fact, just a small representation of it is commonly reported, and most of the compounds found in the literature are highly similar to the original hit, evincing the application of a Free Wilson [26,27,28,29] approach during the discovery process (which assumes an additive and independent contribution of specific substituent groups on the biological activity of a molecule). This methodology, widely used in drug discovery, leads to the exploration of a tiny space surrounding the initial bioactive molecule, leaving a great part of the chemical space unexplored, which may hide potential lead candidates that might surpass the activity or undesired side effects of the original hit. In this sense, the integration of rational selection algorithms in the early drug discovery pipeline could expand the exploration of the chemical space, allowing the selection of more distant representative compounds [10,30,31,32,33,34,35] or guiding repurposing campaigns [36]. This leads one to ask, what would have happened if the authors of a given patent had applied rational selection algorithms to determine which compounds should be synthesized and biologically tested? How much better could they have explored the chemical space? To answer these questions, we have applied diversity selection methods to combinatorial libraries of different sizes, derived from the Markush structure described on the patent of seven new chemical entities approved by the FDA. We have compared the representativeness between the compounds claimed in each patent with reported activity found in the literature and subsets of compounds rationally selected using cluster analysis and partition-based (or partitioning) methods.

## 2. Results and Discussion

### 2.1. Markush Combinatorial Library

To demonstrate that rational selection can improve the efficiency to describe a chemical space, we have studied seven drug patents approved by the FDA (six of them in the last 12 years) referring to different therapeutic targets, with different sizes (in terms of the number of analogs included in the Markush structure) and including on-patent (ONP) and off-patent (OFP) drugs. OFP drugs have been included in the study to discuss whether the exploration of the chemical space is limited due to the situation of their property rights. Accordingly, Leflunomide (the oldest approved drug considered) has been added to the study to verify that this exploratory deficiency is still present in an older patent (see Table 1). 

**Figure 1:**
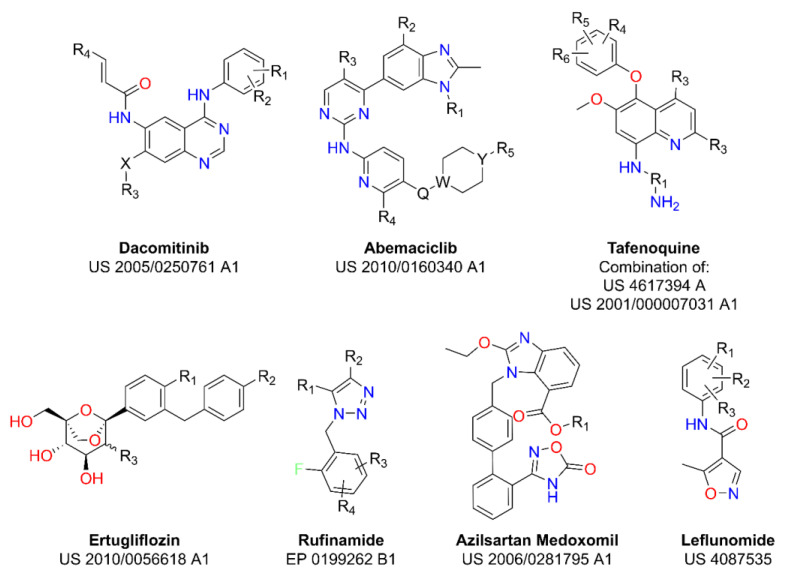
Markush structure reported for the seven drugs under study.

Thus, we have tested the goodness of rational selection over the traditional approach using seven different datasets obtained from the Markush structure stated in the patents of Dacomitinib, Abemaciclib, Tafenoquine, Ertugliflozin, Rufinamide, Azilsartan Medoxomil and Leflunomide drugs [37,38,39,40,41,42,43,44], retrieved from the Orange Book [45]. Corresponding Markush combinatorial libraries (MCL) have been developed from the Markush structure stated in the patent of each drug (Figure 1). The complete list of substituents for each dataset can be found in the Supporting Information (Tables S1–S7).

As expected, some of the Markush structures use vague indeterminations tending to protect vast chemical spaces. As an example, in the present cases of study, the enumeration of Dacomitinib analogs would lead to a large library of more than 25 million compounds, hampering the computational manageability of the dataset. Therefore, in this unique case, the MCL database was reduced to the combination of its explicitly defined structures in the patent (see Section 3).

### 2.2. Bibliographical Database and Bibliographic Combinatorial Library

Bibliographical databases (BD) refer to all the compounds derived from the Markush structure that have been reported as synthesized and, in some cases, biologically tested. To describe all the explored space known until the date for each patent, we have included not only the individual molecules explicitly declared on the patent’s claims but also all ulterior derivatives found in PubChem [46] (even if their declared current application differs from the one described in the original patent). It is worth mentioning that in this study, we have only used publicly available compounds. As an example, the set of 58 Tafenoquine analogs found in PubChem includes the seven molecules claimed in patents [38,42] and 51 molecules reported in the literature (belonging to the same Markush structure) for different applications (27 in the context of malaria [47,48,49,50,51,52], 5 for parasitic diseases [53], 13 as inhibitors of monoamine oxidase A [54] and 6 with undefined biological activity). 

The number of studied compounds in BD sets differs excessively from the size of the MCL library. On the one hand, the low number of derivatives would evince the existence of synthetical limitations that hamper the obtention of more compounds. On the other hand, it would confirm the application of a highly focused exploratory methodology. For this reason, we have studied a second combinatorial database (named Bibliographic Combinatorial Library, BCL) for each patent by combining only the substituents present in BD, aiming to represent the real combinatorial space synthetically accessible until the date.

### 2.3. Clustering Methods for Chemical Space Exploration

Noting that the performance of clustering methods depends on dataset distribution [55,56], eight clustering and two partitioning methods were applied to assess the chemical space described in each patent of study. 

Hierarchical agglomerative clustering (HRC) with a bottom-up approach with six linkage methods (single, complete, median, average, centroid and Ward), K-Means (KMN) and K-Medoids (KMED), binning and optimum variance binning (OV binning) were firstly used to divide the chemical spaces into *k*-optimal clusters in order to see the grade of homogeneity in the population distribution per cluster. The *k*-optimal value was firstly calculated through the average silhouette criterion [57,58,59], using KMN as the standard clustering methodology for all cases. However, no optimal cluster was obtained for all cases as the average silhouette score measured showed a concave curve trend with large maximum values (see Figure S4). This may be caused by the high correlation of the data due to their combinatorial nature and the large size of the datasets, which commonly suffer from the curse of dimensionality and a lack of efficiency. Given the first inconclusive attempts, new standard values commonly used as a rule of thumb in clustering analysis were considered ($\frac{\sqrt{N}}{2}$, $\sqrt{N}$ and $10\cdot \sqrt{N}$) to find the most representative partitioning size to be applied in further comparative discussions.

The case of Tafenoquine’s chemical space (Table 2) serves as an explanatory example to discuss the population distribution using each different size. The aim of our approach was to find the most balanced distribution of data, avoiding as many singletons and hyper-populated clusters as possible, unveiled by high standard deviation values. Results evidenced that the $\sqrt{N}$ clusters seemed to be a good compromise. This size first shows more balanced population frequencies per cluster than $\frac{\sqrt{N}}{2}$, whose results show large values in their standard deviations. Moreover, compared to $10\cdot \sqrt{N}$, $\sqrt{N}$ clustering presented a much lesser number of singletons.

After identifying $\sqrt{N}$ as the best clustering size for our study, the population distribution was assessed for all the clustering methods. HRC single, median and centroid clustering methods were discarded as they tend to represent a unique or a few hyper-populated clusters and a high number of singletons, losing the homogeneous representativeness of the chemical space (Figure S5 in Supporting Information). This distribution is the common result of many hierarchical agglomerative clustering steps with a bottom-up approach. Therefore, HRC average, HRC complete and Ward have been considered as hierarchical agglomerative clustering methodologies, KMN and KMED as non-hierarchical relocation clustering algorithms and OV binning as a representation of cell-based partitioning methods.

### 2.4. Bibliographical Representativeness in Its Chemical Space

To assess the degree of representativeness of BD in the chemical space claimed in a patent (MCL), space and population coverage (SC and PC, respectively) values have been calculated when dividing the MCL space into $N_{BD}$ partitions. Results show that a selection of an equal-sized set using random sampling better represents the chemical space than BD compounds (Table 3 and Table 4).

For example, the 58 molecules of Tafenoquine found in the literature are placed in 10 HRC Ward clusters, leading to 17.2% SC (10 out 58 clusters), and they include 5339 analogs, resulting in 18.8% PC (4789 out 25,472 compounds). In these conditions, even a random selection is able to achieve 61.8% SC and 65.3% PC. This trend is observed regardless of the clustering or partition-based method used and in all the seven cases. Only the application of the HRC Complete cluster algorithm on Azilsartan Medoxomil leads to better results than averaged random selections—although, in this case, results are clearly determined by the low number of compounds to select (*N_BD_* = 4). These results evince that the chemical space claimed in a drug patent is poorly described.

Needless to say, the use of rational selection would always cover 100% of the current partitioned chemical space by selecting one molecule of each cluster. Then, following the abovementioned example, a rational selection of 58 molecules would represent the 100% SC and PC of Tafenoquine’s chemical space. However, this does not imply the synthetical feasibility of the chosen compounds.

### 2.5. Comparing the Chemical Space Described by MCL, BD, and BCL

**Figure 2:**
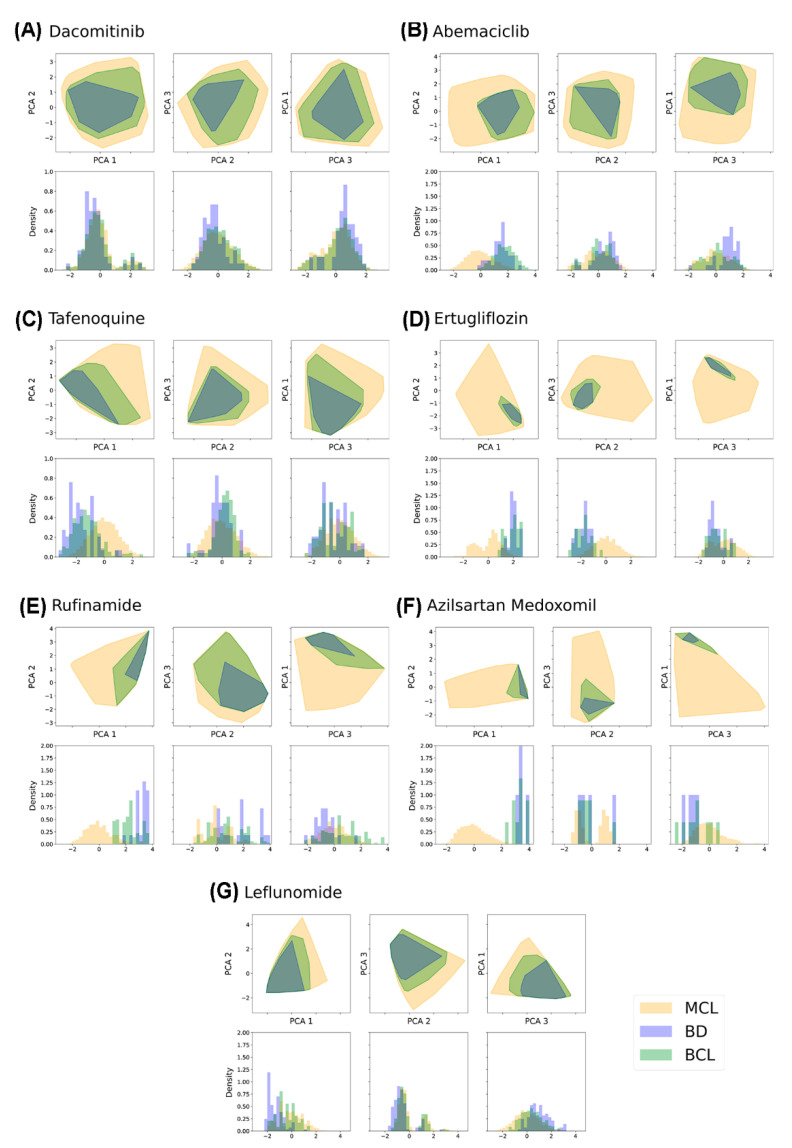
For each subfigure, in the first row, there is the graphical representation of the scatter plot contour of MCL (pale yellow), BD (blue) and BCL (green) libraries when projected on PC1-PC2 (left), PC2-PC3 (middle) and PC3-PC1 (right) planes. In the second row, we show the density histogram for the considered libraries along PC1 (left), PC2 (middle) and PC3 (right). This scheme is repeated for all patents: (**A**) Dacomitinib, (**B**) Abemaciclib, (**C**) Tafenoquine, (**D**) Ertugliflozin, (**E**) Rufinamide, (**F**) Azilsartan Medoxomil and (**G**) Leflunomide.

Quantifying the reduction of the MCL space that implies the use of BCL might be of key importance since its compounds are the ones genuinely expected to be synthetically feasible. This confirmation relies on the fact that BCL compounds include only the fragments coming from real studied candidates, constituting a more legitimate representation of the drug patent. For this purpose, the coverage of the MCL, BD and BCL libraries was compared by assessing their distribution along with each principal component and their density plots when projected on the space of the first three principal components (Figure 2). As expected, BD and BCL progressively improve the description of MCL, although neither of them, except for Dacomitinib and Leflunomide’s analogs (later discussed), significantly cover the chemical space derived from the Markush structure.

To quantify this observation, cluster analysis was performed, setting the number of clusters to a standard size of $\sqrt{N}$. Firstly, according to the results, aligned with the previous study, random selection once again was shown to better represent the overall space rather than compounds derived from the current R&D methodology (BD), even with a higher number of clusters. For the sake of clarity, only results for Tafenoquine and Dacomitinib, using HRC average clustering and OV binning, are shown (Table 5 and Table 6). The results of all the libraries can be found in the Supporting Information (Tables S8–S14).

Although having changed the number of partitions, results agree with the ones described in the previous section: both SC and PC percentages obtained using a random selection are better than those obtained with the bibliographic database, either BD or BCL. Again, bibliographical databases only show better results in the HRC average method applied to Azilsartan Medoxomil, due to its size (Table S13). Overall, contrasting the obtained results with $\sqrt{N}$ and *N_BD_* partitioned space, one may estimate an optimal and synthetically manageable number of samples that could be chosen to be synthesized.

Although the BCL library includes a higher number of compounds, in many cases, it is not able to represent the MCL space; hence, this result would serve as evidence that the synthetically accessible chemical space for each dataset is still poorly known as the rational R&D methodology has followed a mainly focused trend around some original hits. In fact, the use of a rational selection of BD compounds would increase the efficiency of both traditional and cherry-picking methodologies in terms of coverage. As an example, when applying a rational selection of 58 Tafenoquine analogs, an optimal value of 36.3% of SC could be achieved (58 out of 160 clusters).

In contrast to other libraries, Dacomitinib’s BD showed high SC and PC values and a very centered distribution. This is explained by the reduction of the substituents considered in library enumeration (only those in claim 5, see Materials and Methods). This affected not only the MCL size (obtaining 16,530 compounds) but also the BCL (798 compounds). Consequently, the BCL comprises a significant number of analogs of the used dataset of 16,530 compounds. Thus, much lower SC and PC values would be expected when considering the full combinatorial database with more than 25 million analogs.

Additionally, Leflunomide is the unique example in which the number of molecules found BD (*N_BD_*) was greater than $\sqrt{N}$, so the size of the corresponding *BCL* has better representativeness in its chemical space, rather than a random choice of $\sqrt{N}$.

### 2.6. Towards a More Efficient Methodology

**Figure 3:**
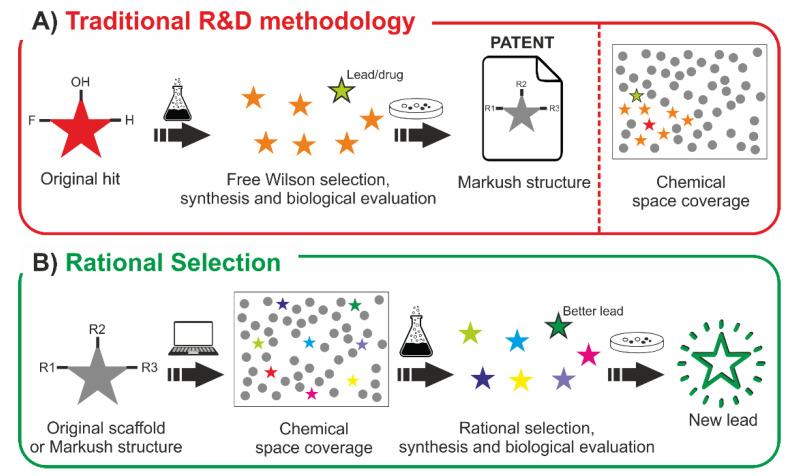
Hit-to-lead process comparative workflow between the traditional methodology (**A**) and the approach suggested by the authors after the reported study (**B**).

In light of the results of our study, enough evidence has been exposed to prove the lack of chemical space exploration in the traditional R&D methodology. This is the result of a procedure that relies on the hit-to-lead optimization step, which commonly aims to find an optimal compound around the original hit, typically involving a Free Wilson approach during the process (Figure 3). The synthesis and biological evaluation of these analogs is performed, leading to a lead or directly a drug candidate and, accordingly, a Markush structure is settled, defending that its involved analogs may present the same biological behavior as the original hit. In fact, even the fragment combination of the reported structures (BCL) does not represent properly the drug’s chemical space derived from the Markush structure (MCL).

**Figure 4:**
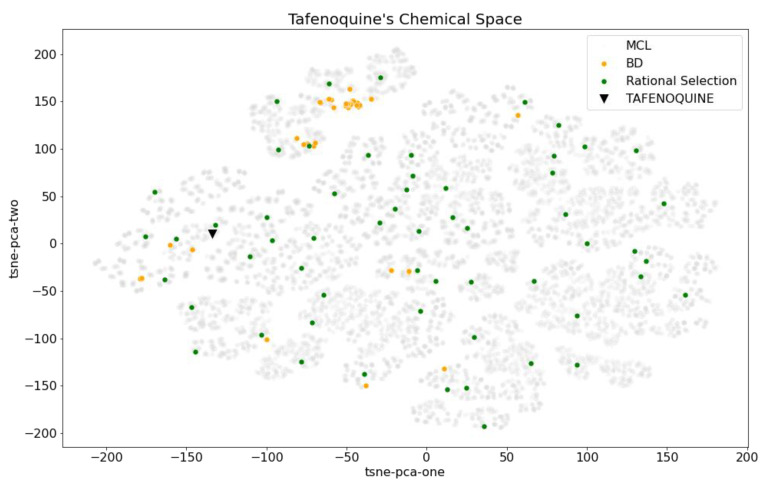
*t*-SNE representation of Tafenoquine’s chemical space, depicting the MCL compounds (grey), BD subset (orange), compounds selected by the rational selection of *N_BD_* analogs (green) and the original hit (black triangle).

This fact can be observed in the aforementioned example, considering the division of Tafenoquine’s chemical space in 58 (*N_BD_*) HRC Ward clusters. BD compounds are located in focused regions of the chemical space, while the rational selection is spread throughout it (Figure 4), leading to a better representation of the whole space.

Hence, we defend an alternative or complementary approach that departs from the combinatorial library obtained from a theoretical Markush structure or from the fragment combination of and original scaffold explored in previous studies (which would ensure the synthetical feasibility of its analogs). Secondly, a computational study, involving space clustering or partitioning, is suggested to rationally choose a handleable number of compounds to synthesize and test that may unveil a better lead in unexplored regions. Thus, this methodology would better consider the chemical diversity of the original Markush set, being the rational selected compounds a significant representation for the issue of study or further repurposing approaches.

## 3. Materials and Methods

### 3.1. Enumeration of Combinatorial Libraries

Markush combinatorial libraries (MCL) were fully enumerated for all patents except for Dacomitinib, which was reduced to a computationally handleable size by only combining the fragments present in the 51 compounds described in claim 5 (including all the positional substitutions in the aryl ring, reducing the more than 25 million original compounds to 19 × 15 × 58 = 16,530). On the contrary, in the antimalarial Tafenoquine case, the library of analogs was extended to the combination of the structures found in several expired patents that included this drug. The BCL databases were further prepared following the protocol described.

MarvinSketch was used for drawing, displaying and enumerating the Markush analogs of the chemical libraries (Marvin 20.21, 2020, ChemAxon Ltd.) [60].

The PubChem search has been performed programmatically by using the PUG-REST [61] Application Programming Interface (API). The whole procedure was integrated into a Python script, which checked the presence of a given compound (entered as a SMILES) on PubChem.

### 3.2. Describing the Chemical Space

All databases were desalted, protonated at pH 7, and their partial charges were calculated using the MMFF94x forcefield in MOE2020.09 software [62]. A total of 206 1D and 2D molecular descriptors were calculated using MOE2020.09 (the complete list of molecular descriptors is available in the Supporting Information, Table S15) to describe the chemical space mathematically. For all combinatorial libraries derived from each Markush structure (MCL libraries), the space dimensionality was reduced using Principal Component Analysis (PCA), keeping 95% of the original variance. The PCA was performed through Python scripting using Scikit-Learn [63].

### 3.3. Clustering and Partitioning Methodologies

The selection of diverse subsets was performed by comparing cluster analysis and partition-based methods on the PCA chemical space. Hierarchical agglomerative clustering with a bottom-up approach (HRC) was performed using the Scikit-Learn toolkit [63], considering Euclidean metrics and assessing different linkage criteria (single, complete, median, average, centroid and Ward). The K-means (KMN) and K-medioids (KMED) non-hierarchical clustering relocation algorithms were performed using the Scikit-Learn [63] and pyclust toolkits, respectively (pyclust PyPI. available online: https://pypi.python.org/pypi/pyclust/0.2.0, accessed on 10 July 2021). To assess the goodness of partition-based methods, binning and optimum variance binning (OV binning) were implemented in Python, adapting the procedure reported by Pascual et al. [22] (see Supporting Information, Figures S1–S3).

### 3.4. Space and Population Coverage

The molecular representativeness and space description of selected diverse subsets were assessed using two standard parameters: the space coverage and the population coverage [64,65].

On the one hand, space coverage (SC) represents the percentage of selected (occupied) clusters or bins (k_oc_) by a given number of selected compounds over the total number of partitions, K (Equation (1)).

(1) $$\frac{k_{oc}}{K}\cdot 100$$

On the other hand, given a database of N compounds, population coverage (PC) measures the SC weighted by the occupancy in each cluster or bin, by dividing the population of the occupied clusters (*n_oc_*) among N (Equation (2)).

(2) $$\frac{n_{oc}}{N}\cdot 100$$

**Figure 5:**
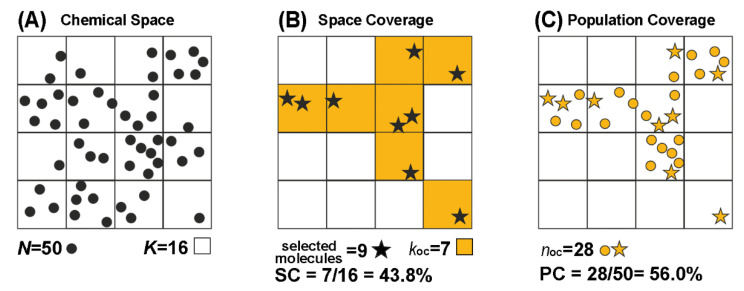
Considering the selection of 9 compounds among a database consisting of 50 molecules (*N* = 50, represented as dots). (**A**) After applying a clustering or partition-based method, the chemical space is divided into 16 partitions (clusters or occupied bins, *K* = 16). (**B**) If the 9 selected compounds (depicted as stars) are distributed in 7 clusters (*k_oc_* = 7, in orange), this corresponds to 43.8% SC. (**C**) Moreover, in these colored clusters are involved 28 samples of the database (*n_oc_*). Hence, the 7 selected molecules are representative of 28 out of the 50 compounds included in the database, which represents 56.0% PC.

The procedure to calculate SC and PC is exemplified in Figure 5.

### 3.5. Number of Clusters

Different numbers of clusters have been studied in the present work to assess the chemical space in terms of population and space coverage. Actually, the number of partitions (whether they are clusters or bins) ultimately determines the number of compounds to pick via rational selection, since they will correspond to the representative compound from each partition.

Preliminary studies were carried out to assess the behavior of a *k*-optimal, $\frac{\sqrt{N}}{2}$, $\sqrt{N}$ and $10\cdot \sqrt{N}$ space fragmentation, being N the total number of compounds in each dataset. Finally, $\sqrt{N}$ was taken as a reference in clustering to optimally represent large datasets. Moreover, BD and BCL sizes (*N_BD_* and *N_BCL_*, correspondingly) were also used to discuss the representativeness of the analogs known until the date, in contrast with a random selection of the same size. Random selections were calculated as the mean value of the coverages represented by *N_BD_* and *N_BCL_* random samples (with 5000 repetitions) as a contrasting result with data found in the bibliography or other rational selections.

## 4. Conclusions

The hit-to-lead process in drug discovery has been traditionally based on the application of the Free Wilson approach, according to which, after hit identification, the structure of the drug candidate is progressively modified, attempting to improve its biological activity. Hence, the resulting procedure allows for exploring the surrounding chemical space of the initial hit compound, but there could be regions that remain unexplored, compromising the R&D efficiency. 

We have assessed how well the chemical space claimed in a patent is actually explored, using seven patents as examples. For all cases, the space explored in the literature (BD) for each combinatorial library is very small, approximately 20% on average when clustering the chemical space in $\sqrt{N}$ clusters. Moreover, results show that even a random selection (by cherry-picking) may lead to better coverage than the molecules reported in the literature. These results are in agreement with results previously reported by our research group regarding the study of the chemical space described by HEPT analogs [22,23].

It has also been evidenced that, in most cases (5 out of 7), even the synthetically accessible combinatorial library (BCL), resulting from the fragmental combination of the molecules described in the literature, is not entirely representative of the chemical space (with lower values than a random study of $\sqrt{N}$ molecules).

Neither the real space explored (BD) nor the fragment combination of the studied molecules (BCL) significantly represents the space defined by the combinatorial libraries derived from the Markush structure, especially when they are compared with the coverage obtained by a statistically random sampling of $\sqrt{N}$ molecules. Thus, there is a large part of the chemical space claimed on patents that remains unexplored, and it can hide potential leads that may surpass the activity of the original hit or reduce undesired side effects. Rational selection algorithms could assist the traditional methodology to optimize the selection of representative compounds of a given chemical space. This could be applied to explore many pharmacokinetic profiles, such as toxicity, biological activity or solubility, among others.

Results reinforce the proposal to integrate the rational selection in the R&D process in early drug discovery or combine it in a mixed methodology involving a local optimization around the original hit. Nevertheless, it should be noted that many large libraries derived from a Markush structure may present candidates with problematic or even unfeasible synthesis and, hence, proper data curation is mandatory before proceeding to a definitive rational selection.

## Figures and Tables

**Table 1:** Information related to drugs under study. The year of approval (Year), the name of the patent applicant (Applic), the name of the disease (Disease), the patent status (Status), the number of analogs included in MCL (*N*), in Bibliographical Data (*N_BD_*) and in Bibliographical Combinatorial Library (*N_BCL_*) and the root square of *N*.

Drug	Year	Applic	Disease	Status	N	$N_{BD}$	$N_{BCL}$	$\sqrt{N}$
**Dacomitinib**	2018	Pfizer Inc.	Metastatic Non-Small-Cell Lung Cancer	ONP	16,530	60	798	129
**Abemaciclib**	2017	Eli Lilly	Breast Cancer	ONP	45,696	41	736	214
**Tafenoquine**	2018	GSK	Malaria	ONP	25,472	58	600	160
**Ertugliflozin**	2017	Merck	Diabetes	ONP	14,194	21	56	120
**Rufinamide**	2008	ESAI	Lennox-Gastaut Syndrome	OFP	8959	22	144	95
**Azilsartan Medoxomil**	2011	Takeda	Hypertension	ONP	1110	4	9	34
**Leflunomide**	1998	Sanofi	Rheumatoid Arthritis	OFP	5641	114	2844	76

**Table 2:** Population distribution per cluster (*k*) analysis in Tafenoquine’s analogs’ chemical space, varying the clustering standard sizes. The mean value ($\bar{x}$) of the population frequencies per cluster and its subsequent standard deviation (σ), along with the ratio of singletons to number of clusters (*%s*), are considered in discussion.

	$k=\frac{\sqrt{N}}{2}=80$		$k=\sqrt{N}=160$		$k=10\cdot \sqrt{N}=1600$	
	$\bar{x}\pm \sigma$	%s	$\bar{x}\pm \sigma$	%s	$\bar{x}\pm \sigma$	%s
HRC single	318 ± 2582	28.7	159 ± 1816	26.2	16 ± 63	18.1
HRC complete	318 ± 184	0.0	159 ± 93	0.0	16 ± 11	0.0
HRC median	318 ± 847	0.0	159 ± 333	0.0	16 ± 23	5.7
HRC average	322 ± 348	0.0	159 ± 151	0.0	16 ± 14	0.7
HRC centroid	322 ± 2294	7.6	159 ± 1436	6.9	16 ± 25	7.4
HRC Ward	318 ± 113	0.0	159 ± 54	0.0	16 ± 6	0.0
KMN	318 ± 72	0.0	159 ± 38	0.0	16 ± 5	0.0
KMED	318 ± 125	0.0	159 ± 64	0.0	16 ± 7	0.2
Binning	411 ± 269	0.0	209 ± 165	0.0	20 ± 25	10.8
OV binning	509 ± 555	0.0	173 ± 201	0.7	20 ± 25	9.8

**Table 3:** Comparison of SC and PC values obtained by random selection and BD compounds when dividing the MCL of each patent in *N_BD_* clusters for Dacomitinib, Abemacilib and Tafenoquine databases.

		*Dacomitinib*		*Abemaciclib*		*Tafenoquine*	
		BD	Random	BD	Random	BD	Random
**HRC average**	**SC**	26.7	41.1	22.0	35.1	17.2	46.8
	**PC**	74.2	80.1	66.2	85.9	12.8	78.4
**HRC** **Complete**	**SC**	50.0	54.0	34.1	52.0	25.9	59.3
	**PC**	69.0	72.8	44.9	74.0	19.3	67.6
**HRC Ward**	**SC**	41.7	53.5	34.1	58.6	17.2	61.8
	**PC**	71.5	73.2	43.9	68.8	18.8	65.3
**KMN**	**SC**	48.3	57.6	34.1	60.3	22.4	62.5
	**PC**	67.3	69.4	39.1	67.4	21.0	64.7
**KMED**	**SC**	55.0	57.6	34.1	59.1	24.1	61.4
	**PC**	68.0	68.8	44.4	68.3	21.8	65.9
**OV binning**	**SC**	25.0	42.9	28.1	51.8	24.0	49.2
	**PC**	59.1	78.3	46.2	84.0	35.4	82.9

**Table 4:** Comparison of SC and PC values obtained by random selection and BD compounds when dividing the MCL of each patent in *N_BD_* clusters for Ertugliflozin, Rufinamide, Azilsartan Medoxomil and Leflunomide databases.

		*Ertugliflozin*		*Rufinamide*		*Azilsartan* *Medoxomil*		*Leflunomide*	
		BD	Random	BD	Random	BD	Random	BD	Random
**HRC average**	**SC**	9.5	19.8	36.4	30.6	50.0	26.4	27.2	50.1
	**PC**	83.8	88.4	6.4	86.2	1.3	98.7	11.6	76.4
**HRC** **Complete**	**SC**	23.8	38.7	22.7	43.6	50.0	26.3	26.3	55.3
	**PC**	59.4	78.8	4.0	79.7	1.3	98.7	17.9	71.5
**HRC Ward**	**SC**	19.0	57.7	13.6	70.8	50.0	41.0	15.8	60.6
	**PC**	26.9	69.7	6.2	67.1	82.7	87.0	15.7	66.8
**KMN**	**SC**	23.8	58.7	18.2	58.9	50.0	52.1	15.8	60.8
	**PC**	23.5	69.3	7.9	69.4	59.9	84.8	13.1	66.7
**KMED**	**SC**	19.0	57.7	27.3	61.6	50.0	68.3	18.4	59.8
	**PC**	29.8	70.8	27.4	66.6	46.8	68.8	20.8	67.6
**OV binning**	**SC**	13.3	65.6	28.6	50.3	25.0	56.6	26.4	53.0
	**PC**	18.2	82.2	9.6	90.1	1.7	80.2	32.5	83.0

**Table 5:** SC and PC results for a number of $k=\sqrt{N}$ clusters for Tafenoquine analogs.

		HRC Average		HRC Complete		*OV Binning*	
*Tafenoquine*	Selection Size	*SC*	*PC*	*SC*	PC	SC	PC
**BD**	58	8.8	6.4	11.9	9.7	13.6	17.0
**Random BD**	58	27.0	45.4	29.1	37.1	27.1	53.6
**BCL**	600	38.8	33.2	33.8	28.3	36.0	49.3
**Random BCL**	600	80.4	95.6	90.6	96.1	74.7	96.1
**Random (** $\sqrt{N}$ **)**	160	50.9	74.8	57.7	69.1	48.2	79.8

**Table 6:** SC and PC results for a number of $k=\sqrt{N}$ clusters for Dacomitinib analogs.

		HRC Average		HRC Complete		*OV Binning*	
*Dacomitinib*	Selection size	*SC*	*PC*	*SC*	PC	SC	PC
**BD**	60	20.9	50.3	27.1	42.1	16.8	52.0
**Random BD**	60	31.2	54.8	34.4	46.4	30.2	70.6
**BCL**	798	79.1	92.1	86.8	90.9	64.3	92.3
**Random BCL**	798	86.9	98.4	95.9	98.8	81.9	98.1
**Random (** $\sqrt{N}$ **)**	129	49.4	74.7	56.2	69.9	45.1	83.5

## Data Availability

Data are contained within the article and supplementary material.

## Supplementary Materials

The following supporting information can be downloaded at: https://www.mdpi.com/article/10.3390/ph15091159/s1, Figure S1: Description of the Binning and OV Binning partitioning methods implemented in this study; Figure S2: Application of binning and OV binning on Tafenoquine’s dataset to divide the chemical space into 8 bins, Figure S3: OV Binning partitioning representation of Tafenoquine’s case study dataset for 2, 4, 8, 16, 32 and 64 bins; Figure S4: Silhouette Score Elbow plot for KMN clustering of Tafenoquine database; Figure S5: Boxplots of population distribution for the 6 libraries of analogues and √N clusters; Table S1: Dacomitinib fragment library (16,530 analogues); Table S2: Abemaciclib fragment library (45,696 analogues); Table S3: Tafenoquine fragment library (25,472 analogues); Table S4: Ertugliflozin fragment library (14,194 analogues); Table S5: Rufinamide fragment library (8959 analogues); Table S6: Azilsartan Medoxomil fragment library (1110 analogues); Table S7: Leflunomide fragment library (5641 analogues); Table S8: SC and PC for Dacomitinib analogues ($k=\sqrt{N}$); Table S9: SC and PC for Abemaciclib analogues ($k=\sqrt{N}$); Table S10: SC and PC for Tafenoquine analogues ($k=\sqrt{N}$); Table S11: SC and PC for Ertugliflozin analogues ($k=\sqrt{N}$); Table S12: SC and PC for Rufinamide analogues ($k=\sqrt{N}$); Table S13: SC and PC for Azilsartan Medoxomil analogues ($k=\sqrt{N}$); Table S14: SC and PC for Leflunomide analogues ($k=\sqrt{N}$); Table S15: List of 206 1D and 2D descriptors calculated using MOE2020.09.
