# Supplementary material for: Deconstructing Markush: Improving the R&D Efficiency Using Library Selection in Early Drug Discovery
Source: Pharmaceuticals (Basel). 2022 Sep 18;15(9):1159. doi: 10.3390/ph15091159 (PMC9503783; doi:10.3390/ph15091159)
Supplement: Supplementary file 1 [file pharmaceuticals-15-01159-s001.zip › pharmaceuticals-1837185-supplementary.pdf]

**Supporting Information**

# Deconstructing Markush: Improving the R&D efficiency using Library Selection in Early Drug Discovery

*Leticia Manen-Freixa, José I. Borrell, Jordi Teixido Closa, Roger Estrada-Tejedor**

Molecular Design Laboratory, Group of Pharmaceutical Chemistry, IQS School of Engineering, Universitat Ramon Llull, Via Augusta 390, 08017 Barcelona, Spain.

* Corresponding Author: roger.estrada@iqs.url.edu

## Contents

**Figure S1**. Description of the Binning and OV Binning partitioning methods implemented in this study ........ 3

**Figure S2.** Application of binning and OV binning on Tafenoquine's dataset to divide the chemical space into 8 bins. ........ 4

**Figure S3.** OV Binning partitioning representation of Tafenoquine's case study dataset for 2, 4, 8, 16, 32 and 64 bins. ........ 5

**Figure S4**. Silhouette Score Elbow plot for KMN clustering of Tafenoquine database ........ 5

**Figure S5.** Boxplots of population distribution for the 6 libraries of analogues and $\sqrt{N}$ clusters ........ 6

**Table S1.** Dacomitinib fragment library (16,530 analogues). ........ 7

**Table S2.** Abemaciclib fragment library (45,696 analogues) ........ 8

**Table S3.** Tafenoquine fragment library (25,472 analogues) ........ 9

**Table S4.** Ertugliflozin fragment library (14,194 analogues) ........ 10

**Table S5.** Rufinamide fragment library (8,959 analogues) ........ 13

**Table S6.** Azilsartan Medoxomil fragment library (1,110 analogues) ........ 14

**Table S7.** Leflunomide fragment library (5,641 analogues) ........ 15

**Table S8.** SC and PC for Dacomitinib analogues ($k = \sqrt{N}$). ................................................16
**Table S9.** SC and PC for Abemaciclib analogues ($k = \sqrt{N}$). ................................................16
**Table S10**. SC and PC for Tafenoquine analogues ($k = \sqrt{N}$). ..............................................17
**Table S11**. SC and PC for Ertugliflozin analogues ($k = \sqrt{N}$). ..............................................17
**Table S12**. SC and PC for Rufinamide analogues ($k = \sqrt{N}$). ................................................18
**Table S13**. SC and PC for Azilsartan Medoxomil analogues ($k = \sqrt{N}$). ...............................18
**Table S14** SC and PC for Leflunomide analogues ($k = \sqrt{N}$). ................................................19
**Table S15.** List of 206 1D and 2D descriptors calculated using MOE2020.09 ......................20

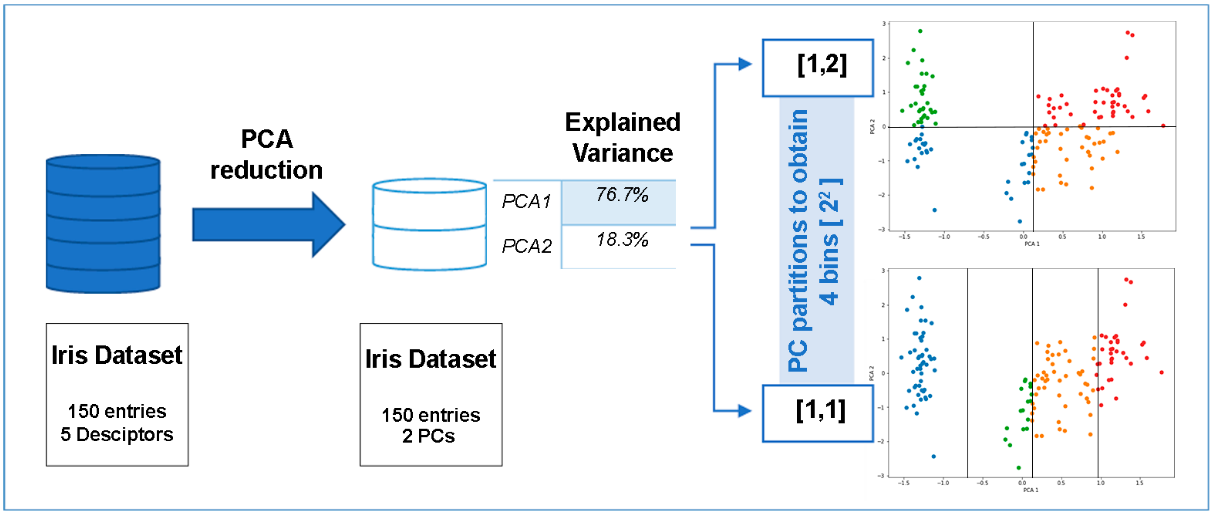


**Figure S1.** Description of the Binning and OV Binning partitioning methods implemented in this study. Fisher's Iris dataset (Fisher, R. A. Ann. Eugen. 1936, 7 (2), 179–188) is used in the example to show how (a) binning partitioned the space in the two firsts PCs while *(b)* OV Binning divided twice the first PCA due to the great variance present in it. **Simple Binning** algorithm approach sorts the Principal Components axes (PCAs) by descending order of its explained variance. When a certain number of bins is requested (*k*), the algorithm will divide each axis subsequently until the number of occupied bins are equal or less than *k*. Hence, each partition results in the formation of $2^n$ bins, being *n* the number of divided PCs. **Optimum Variance Binning** (OV Binning) algorithm is a modification of the Optimum Binning algorithm described by Pascual *et.al.* (Mol. Divers. 2003, 6 (2), 121–133). It also sorts the PCAs by descending order of its explained variance but, assuming that the division of an axis divides its variance in two subaxes, the following partition is reimplemented in the same PCA only if its variance is greater than the explained variance of the following axis. With this procedure, multiple partitions can be observed in the first PCAs as they contain higher explained variance values proceeding from the Principal Component Analysis. Hence, molecules are binned with a bias toward the PCs that exhibit the largest variation (see Figures S2 and S3).

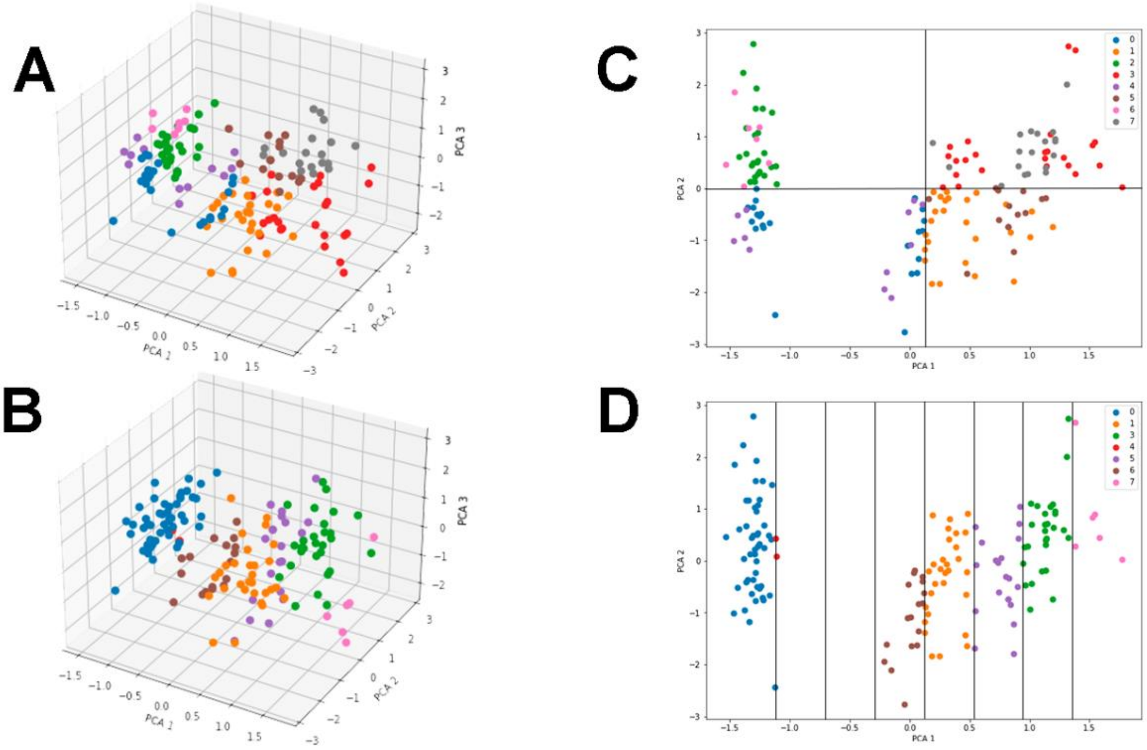


**Figure S2.** Application of binning and OV binning on Tafenoquine's dataset to divide the chemical space into 8 bins. The figure shows the representation of the 8 bins in 3D (A, B) and 2D (C, D). For 2D examples, partition subaxes are represented when the showed axes are divided: once for PCA1, PCA2 and the non-represented PCA3 in binning (C) and three times for PCA1 in OV binning (D).

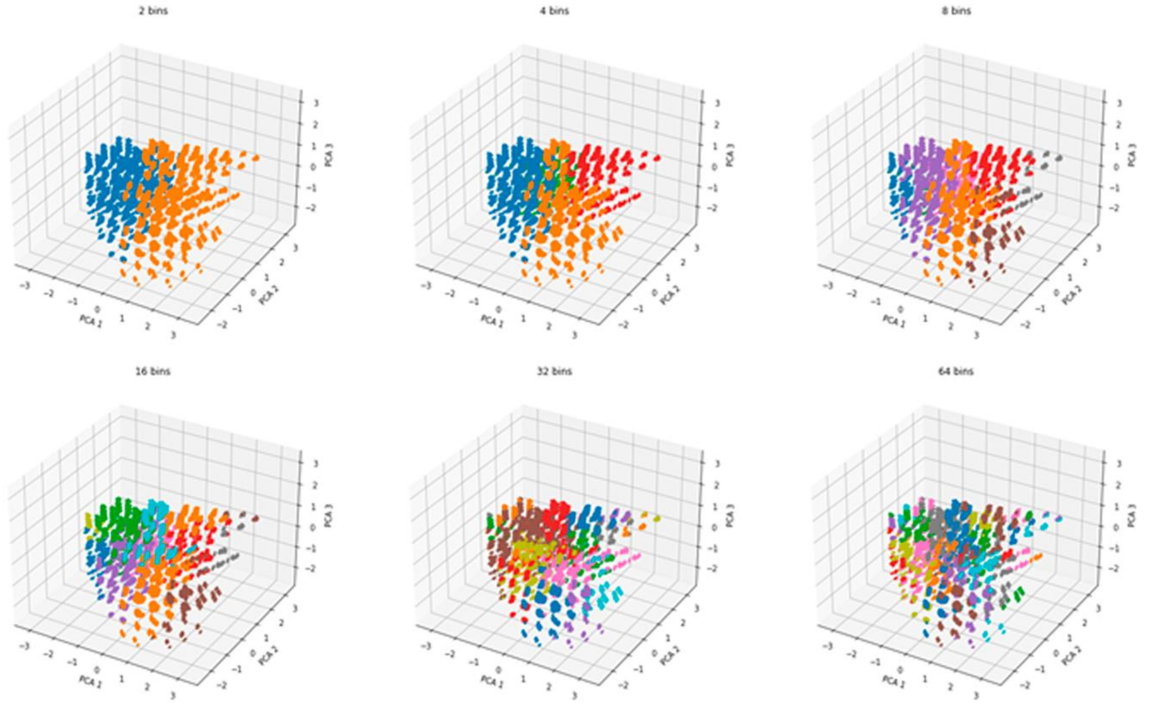


**Figure S3.** OV Binning partitioning representation of Tafenoquine's case study dataset for 2, 4, 8, 16, 32 and 64 bins.

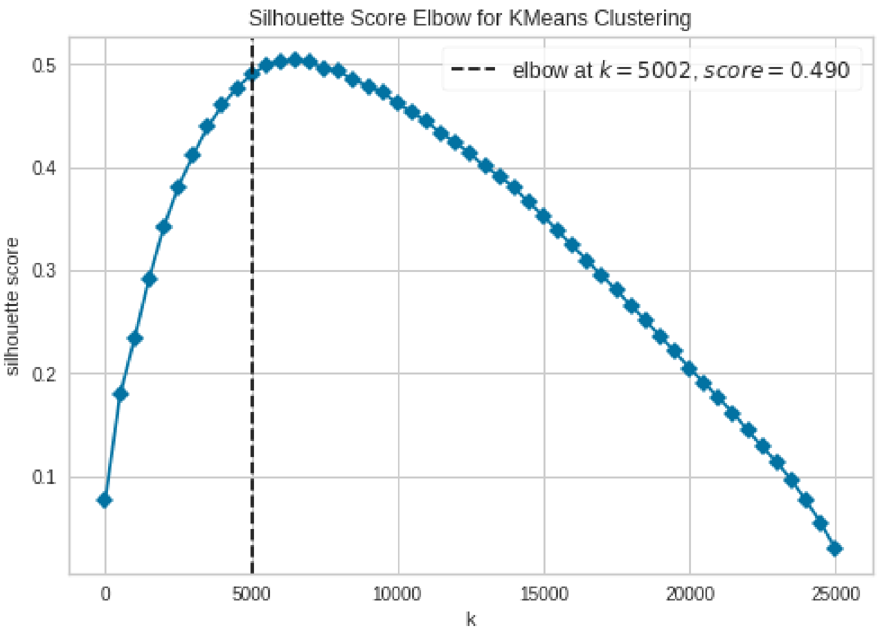


**Figure S4.** Silhouette Score Elbow plot for KMN clustering of Tafenoquine database.

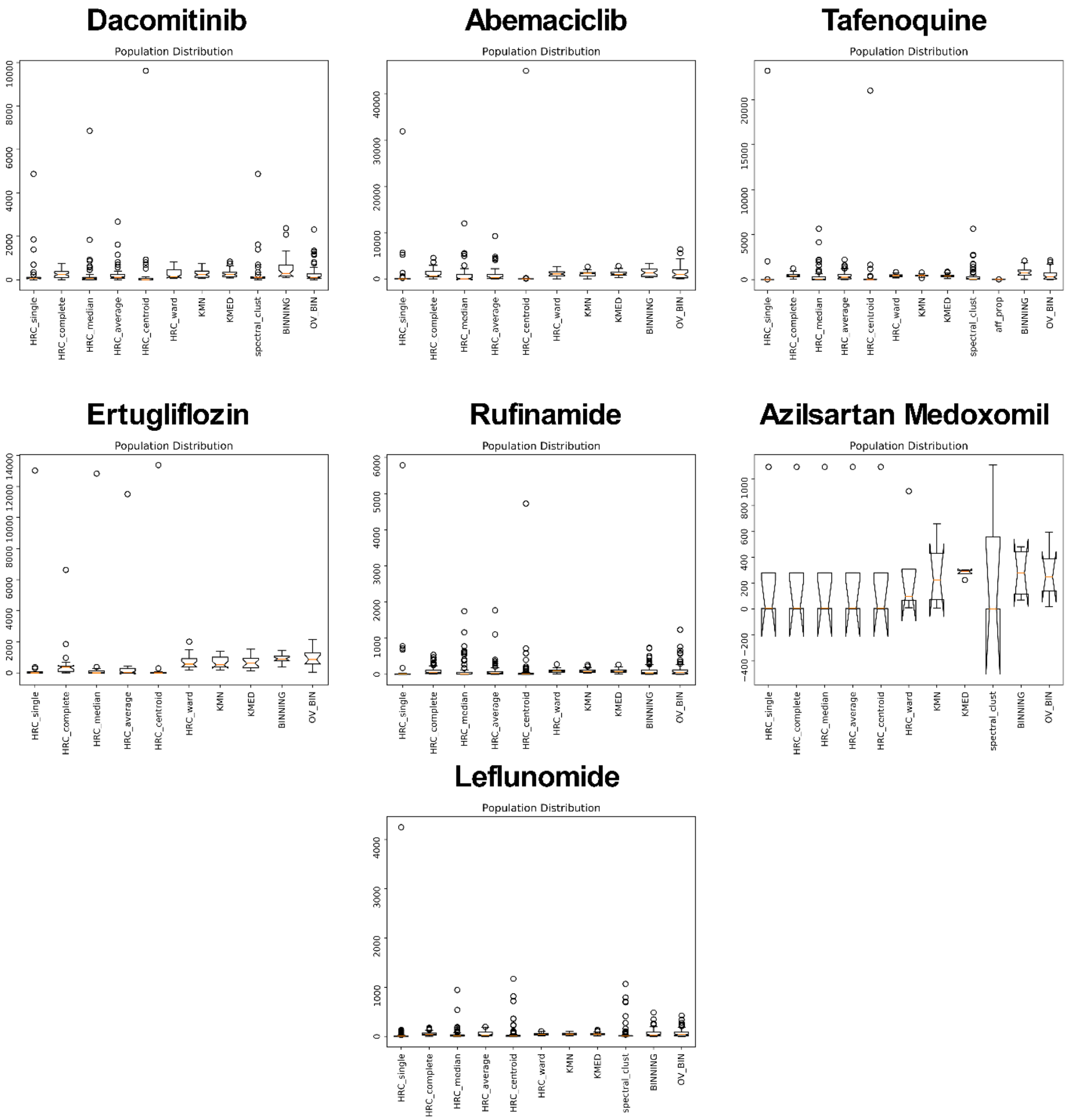


**Figure S5.** Boxplots of population distribution for the 6 libraries of analogues and $\sqrt{N}$ clusters.

**Table S1.** Dacomitinib fragment library (16,530 analogues).

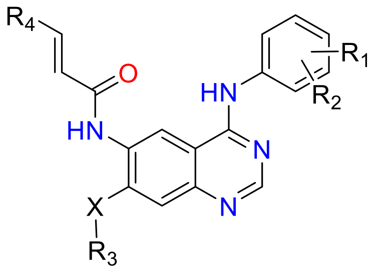


| R1 | R2 | X | R3 | R4 |
|---|---|---|---|---|
| -H | -H | -O- | -C | -CN1CCCCC1 |
| -F | -F | -S- | -CC | -CN1CCC(F)CC1 |
| -Br | -Br | -N(H)- | -CCC | -CN1CCCC(F)C1 |
| -Cl | -Cl | | -C(C)C | -CN1C(F)CCCC1 |
| | | | -C(F)(F)F | -CN1CCOCC1 |
| | | | -CF | -CN1CCCCCC1 |
| | | | -CCF | -CN1C=CCCC1 |
| | | | -CC(F)(F)F | -CN1CC=CCC1 |
| | | | -C(F)CF | -CN1CCNCC1 |
| | | | -CCCN1CCOCC1 | -CN1CCN(C)CC1 |
| | | | -CCN1CCCCC1 | -Cn1cncc1 |
| | | | | -CN1CCCC1 |
| | | | | -CCN1CCCCC1 |
| | | | | -CCN1CCC(F)CC1 |
| | | | | -CCN1CCCC(F)C1 |
| | | | | -CCN1C(F)CCCC1 |
| | | | | -CCN1CCOCC1 |
| | | | | -CCN1CCCCCC1 |
| | | | | -CCCN1CCCCC1 |

**Table S2.** Abemaciclib fragment library (45,696 analogues).

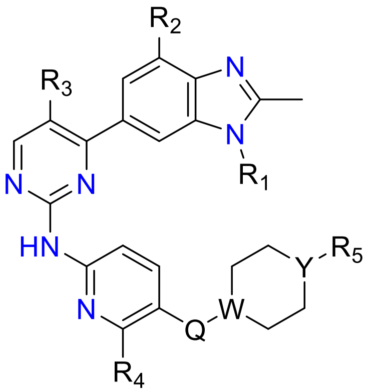


| R1 | R2 | R3 | R4 | Q | W | Y | R5 | R6 | R7 |
|---|---|---|---|---|---|---|---|---|---|
| -CCC | -H | -H | -H | -C- | -C= | -C= | -C | -C | -C |
| -C(C)C | -F | -F | -C | -O- | -N= | -N= | -CC | -CC | -CC |
| -CCCC | | | | -S- | | | -CCC | -CCC | -CCC |
| -C(C)CC | | | | | | | -CCCC | -C(C)C | -C(C)C |
| -CC(C)C | | | | | | | -N(R6)(R7) | -CCCC | -CCCC |
| -C(C)(C)C | | | | | | | | -C(C)(C)C | -C(C)(C)C |
| -CCCCC | | | | | | | | -CCCCC | -CCCCC |
| -CCC(C)C | | | | | | | | -CCCCCC | -CCCCCC |
| -C(C)CCC | | | | | | | | -CCC(C)C | -CCC(C)C |
| -CC(C)CC | | | | | | | | -CC(C)(C)C | -CC(C)(C)C |
| -C(C)C(C)C | | | | | | | | | |
| -C(CC)CC | | | | | | | | | |
| -C1C(C)C1 | | | | | | | | | |
| -C1CC1 | | | | | | | | | |
| -CC1CC1 | | | | | | | | | |
| -C1CCCC1 | | | | | | | | | |
| -C1CCC1 | | | | | | | | | |

**Table S3.** Tafenoquine fragment library (25,472 analogues).

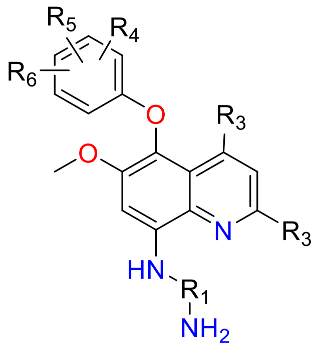


| R1 | R2 | R3 | R4 | R5 | R6 |
|---|---|---|---|---|---|
| -C(C)CCC- | -H | -H | -H | -H | -H |
| -C(C)CCCC- | -C | -OC | -Cl | -Cl | -Cl |
| -C(CC)CCC- | | | -Br | -Br | -Br |
| -C(CC)CCCC- | | | -F | -F | -F |
| -CCCC(C)- | | | -C(F)(F)F | -C(F)(F)F | -C(F)(F)F |
| -CCCCC(C)- | | | -OC | -OC | -OC |
| -CCCC(CC)- | | | | | |
| -CCCCC(CC)- | | | | | |

**Table S4.** Ertugliflozin fragment library (14,194 analogues).

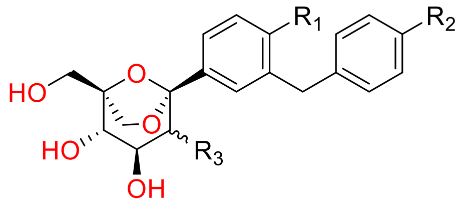


| R1 | R2 | R3 | R4 | R5 |
|---|---|---|---|---|
| -H | -C | -[C@H](O) | -O- | -O- |
| -C | -CC | -[C@@H](O) | -N(H)- | -N(H)- |
| -CC | -CCC | | -S- | -S- |
| -CCC | -C(C)C | | | |
| -C(C)C | -C(C)(C)C | | | |
| -C(C)(C)C | -C(C)CC | | | |
| -C(C)CC | -CCCC | | | |
| -CCCC | -OC | | | |
| -OC | -OCC | | | |
| -OCC | -OCCC | | | |
| -OCCC | -OC(C)C | | | |
| -OC(C)C | -OC(C)(C)C | | | |
| -OC(C)(C)C | -OC(C)CC | | | |
| -OC(C)CC | -OCCCC | | | |
| -OCCCC | -C#C | | | |
| -C#C | -C#CC | | | |
| -C#CC | -CC#C | | | |
| -CC#C | -C#CCC | | | |
| -C#CCC | -CC#CC | | | |
| -CC#CC | -CCC#C | | | |
| -CCC#C | -C(C)C#C | | | |

| | | | | |
|---|---|---|---|---|
| -C(C)C#C<br>-Cl<br>-F<br>-C(F)(F)F<br>-CCF<br>-C(C)F<br>-CN<br>-S(=O)(=O)C<br>-S(=O)(=O)CC<br>-S(=O)(=O)CCC<br>-S(=O)(=O)C(C)C<br>-S(=O)(=O)CCCC<br>-S(=O)(=O)C(C)CC<br>-S(=O)(=O)CC(C)C<br>-S(=O)(=O)C(C)(C)C<br>-C1CC1<br>-C1CCC1<br>-C1CCCC1<br>-C1CCCCC1 | -Cl<br>-F<br>--C(F)(F)F<br>-CCF<br>-C(C)F<br>-CN<br>-S(=O)(=O)C<br>-S(=O)(=O)CC<br>-S(=O)(=O)CCC<br>-S(=O)(=O)C(C)C<br>-S(=O)(=O)CCCC<br>-S(=O)(=O)C(C)CC<br>-S(=O)(=O)CC(C)C<br>-S(=O)(=O)C(C)(C)C<br>-C1CC1<br>-C1CCC1<br>-C1CCCC1<br>-C1CCCCC1<br>-N1CCCC1<br>-N1R4CCC1<br>-N1CR4CC1<br>-C1R4R5CC1<br>-C1R4CR5C1<br>-C1R4CCR51<br>-C1CR4R5C1<br>-C1R4CCCC1<br>-C1CR4CCC1 | | | |

| | | | | |
|---|---|---|---|---|
| | -C1CCR4CC1<br>-N1R4CCCC1<br>-N1CR4CCC1<br>-N1CCR4CC1<br>-C1R4R5CCC1<br>-C1R4CR5CC1<br>-C1R4CCR5C1<br>-C1R4CCCR51<br>-C1CR4R5CC1<br>-C1CR4CR5C1<br>-OC1COC1<br>-OC1COCC1 | | | |

**Table S5.** Rufinamide fragment library (8,959 analogues).

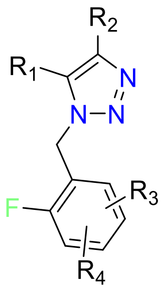


| R1 | R2 | R3 | R4 |
|---|---|---|---|
| -H | -H | -H | -H |
| -C(=O)N | -C(=O)N | -F | -F |
| -C(=O)NC(=O)C | -C(=O)NC(=O)C | -Cl | -Cl |
| -C(=O)NC(=O)CC | -C(=O)NC(=O)CC | | |
| -C(=O)NC(=O)CCC | -C(=O)NC(=O)CCC | | |
| -C(=O)NC(=O)C(C)C | -C(=O)NC(=O)C(C)C | | |
| -C(=O)NC(=O)CCCC | -C(=O)NC(=O)CCCC | | |
| -C(=O)NC(=O)C(C)CC | -C(=O)NC(=O)C(C)CC | | |
| -C(=O)NC(=O)CC(C)C | -C(=O)NC(=O)CC(C)C | | |
| -C(=O)NC(=O)C(C)(C)C | -C(=O)NC(=O)C(C)(C)C | | |
| -C(=O)N(CC)CC | -C(=O)N(CC)CC | | |
| -C(=O)N(CCC)CCC | -C(=O)N(CCC)CCC | | |
| --C(=O)N(CCCC)CCCC | --C(=O)N(CCCC)CCCC | | |
| -C(=O)N(C)C | -C(=O)N(C)C | | |
| -C(=O)N(C(C)C)C(C)C | -C(=O)N(C(C)C)C(C)C | | |
| -C(=O)N(C(C)CC)C(C)CC | -C(=O)N(C(C)CC)C(C)CC | | |
| -C(=O)N(C(C)(C)C)C(C)(C)C | -C(=O)N(C(C)(C)C)C(C)(C)C | | |

**Table S6.** Azilsartan Medoxomil fragment library (1,110 analogues).

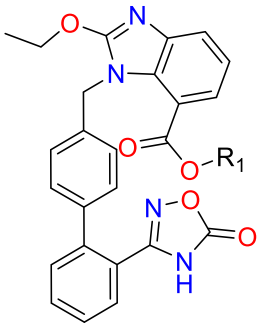


| R1 | R2 | R3 | R4 |
|---|---|---|---|
| -CC1=C(R2)OC(=O)O1 | -C | -C | -C |
| -C1(R2)C(R3)(R4)OC(=O)O1 | -CC | -CC | -CC |
| -C1(R2)C(R3)(R4)CC(=O)O1 | -CCC | -CCC | -CCC |
| | -C(C)C | -C(C)C | -C(C)C |
| | -CCCC | -CCCC | -CCCC |
| | -C(C)CC | -C(C)CC | -C(C)CC |
| | -CC(C)C | -CC(C)C | -CC(C)C |
| | -CC(C)(C)C | -CC(C)(C)C | -CC(C)(C)C |
| | -C(C)(C)C | -C(C)(C)C | -C(C)(C)C |
| | -CCCCC | -CCCCC | -CCCCC |
| | -CCC(C)C | -CCC(C)C | -CCC(C)C |
| | -CC(C)(C)C | -CC(C)(C)C | -CC(C)(C)C |
| | -CCCCCC | -CCCCCC | -CCCCCC |

**Table S7.** Leflunomide fragment library (5,641 analogues).

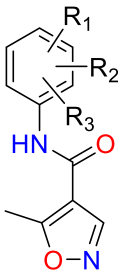


| R1 | R2 | R3 |
|---|---|---|
| -H | -H | -H |
| -F | -F | -F |
| -Cl | -Cl | -Cl |
| -Br | -Br | -Br |
| -I | -I | -I |
| -C(F)(F)F | -C(F)(F)F | -C(F)(F)F |
| -OC | -OC | -OC |
| -COC | -COC | -COC |
| -OCC | -OCC | -OCC |
| -C | -C | -C |
| -CC | -CC | -CC |

**Table S8.** SC and PC for Dacomitinib analogues ($k = \sqrt{N}$).

| *Dacomitinib* | *Number of analogues* | *SpaceCov HRC_ave* | *PopCov HRC_ave* | *SpaceCov HRC_complete* | *PopCov HRC_ complete* | *SpaceCov OV_BIN* | *PopCov OV_BIN* |
|---|---|---|---|---|---|---|---|
| **Bibliographic data *(BD)*** | 60 | 20.9 | 50.3 | 27.1 | 42.1 | 16.8 | 52.0 |
| **Random *(BD)*** | 60 | 31.2 | 54.8 | 34.4 | 46.4 | 30.2 | 70.6 |
| **Combinatorial Biblio data *(BCL)*** | 798 | 79.1 | 92.1 | 86.8 | 90.9 | 64.3 | 92.3 |
| **Random *(BCL)*** | 798 | 86.9 | 98.4 | 95.9 | 98.8 | 81.9 | 98.1 |
| **Random (√N)** | 129 | 49.4 | 74.7 | 56.2 | 69.9 | 45.1 | 83.5 |

| *Dacomitinib* | *Number of analogues* | *SpaceCov HRC_ward* | *PopCov HRC_ward* | *SpaceCov KMN* | *PopCov KMN* | *SpaceCov KMED* | *PopCov KMED* |
|---|---|---|---|---|---|---|---|
| **Bibliographic data *(BD)*** | 60 | 29.5 | 38.4 | 27.9 | 35.8 | 31.0 | 43.0 |
| **Random *(BD)*** | 60 | 35.9 | 46.4 | 30.1 | 41.6 | 35.2 | 44.2 |
| **Combinatorial Biblio data *(BCL)*** | 798 | 92.3 | 92.3 | 90.7 | 92.4 | 86.8 | 90.8 |
| **Random *(BCL)*** | 798 | 98.5 | 99.2 | 98.6 | 99.3 | 97.4 | 99.0 |
| **Random (√N)** | 129 | 59.6 | 67.1 | 60.1 | 66.7 | 58.0 | 68.5 |

**Table S9.** SC and PC for Abemaciclib analogues ($k = \sqrt{N}$).

| *Abemaciclib* | *Number of analogues* | *SpaceCov HRC_ave* | *PopCov HRC_ave* | *SpaceCov HRC_complete* | *PopCov HRC_ complete* | *SpaceCov OV_BIN* | *PopCov OV_BIN* |
|---|---|---|---|---|---|---|---|
| **Bibliographic data *(BD)*** | 41 | 4.7 | 63.8 | 9.8 | 9.9 | 15.9 | 23.7 |
| **Random *(BD)*** | 41 | 6.9 | 81.4 | 16.7 | 24.6 | 23.2 | 50.8 |
| **Combinatorial Biblio data *(BCL)*** | 736 | 26.6 | 31.8 | 30.4 | 27.5 | 46.8 | 51.0 |
| **Random *(BCL)*** | 736 | 64.9 | 95.3 | 85.2 | 95.2 | 74.0 | 97.9 |
| **Random (√N)** | 214 | 42.9 | 81.2 | 55.1 | 71.2 | 54.3 | 89.0 |

| *Abemaciclib* | *Number of analogues* | *SpaceCov HRC_ward* | *PopCov HRC_ward* | *SpaceCov KMN* | *PopCov KMN* | *SpaceCov KMED* | *PopCov KMED* |
|---|---|---|---|---|---|---|---|
| **Bibliographic data *(BD)*** | 41 | 10.3 | 10.1 | 10.3 | 11.0 | 10.7 | 14.7 |
| **Random *(BD)*** | 41 | 17.2 | 19.7 | 17.3 | 19.0 | 17.1 | 20.9 |
| **Combinatorial Biblio data *(BCL)*** | 736 | 24.3 | 24.2 | 25.7 | 24.4 | 27.1 | 33.0 |
| **Random *(BCL)*** | 736 | 93.3 | 96.0 | 94.3 | 96.3 | 92.1 | 95.6 |
| **Random (√N)** | 214 | 60.5 | 66.2 | 61.4 | 65.4 | 59.3 | 67.3 |

**Table S10.** SC and PC for Tafenoquine analogues ($k = \sqrt{N}$).

| *Tafenoquine* | *Number of analogues* | *SpaceCov HRC_ave* | *PopCov HRC_ave* | *SpaceCov HRC_complete* | *PopCov HRC_ complete* | *SpaceCov OV_BIN* | *PopCov OV_BIN* |
|---|---|---|---|---|---|---|---|
| **Bibliographic data *(BD)*** | 58 | 8.8 | 6.4 | 11.9 | 9.7 | 13.6 | 17.0 |
| **Random *(BD)*** | 58 | 27.0 | 45.4 | 29.1 | 37.1 | 27.1 | 53.6 |
| **Combinatorial Biblio data *(BCL)*** | 600 | 38.8 | 33.2 | 33.8 | 28.3 | 36.0 | 49.3 |
| **Random *(BCL)*** | 600 | 80.4 | 95.6 | 90.6 | 96.1 | 74.7 | 96.1 |
| **Random (√N)** | 160 | 50.9 | 74.8 | 57.7 | 69.1 | 48.2 | 79.8 |

| *Tafenoquine* | *Number of analogues* | *SpaceCov HRC_ward* | *PopCov HRC_ward* | *SpaceCov KMN* | *PopCov KMN* | *SpaceCov KMED* | *PopCov KMED* |
|---|---|---|---|---|---|---|---|
| **Bibliographic data *(BD)*** | 58 | 7.5 | 7.0 | 9.4 | 8.0 | 10.0 | 8.6 |
| **Random *(BD)*** | 58 | 30.0 | 32.9 | 30.3 | 31.7 | 20.3 | 45.4 |
| **Combinatorial Biblio data *(BCL)*** | 600 | 26.9 | 27.7 | 37.5 | 36.0 | 44.4 | 41.2 |
| **Random *(BCL)*** | 600 | 95.9 | 97.0 | 96.8 | 97.4 | 94.7 | 96.8 |
| **Random (√N)** | 160 | 61.4 | 65.4 | 62.4 | 64.4 | 60.6 | 66.2 |

**Table S11.** SC and PC for Ertugliflozin analogues ($k = \sqrt{N}$).

| *Ertugliflozin* | *Number of analogues* | *SpaceCov HRC_ave* | *PopCov HRC_ave* | *SpaceCov HRC_complete* | *PopCov HRC_ complete* | *SpaceCov OV_BIN* | *PopCov OV_BIN* |
|---|---|---|---|---|---|---|---|
| **Bibliographic data *(BD)*** | 21 | 8.3 | 12.2 | 6.7 | 9.2 | 6.8 | 7.8 |
| **Random *(BD)*** | 21 | 13.4 | 41.5 | 14.7 | 25.4 | 20.2 | 51.0 |
| **Combinatorial Biblio data *(BCL)*** | 56 | 12.5 | 17.7 | 10.8 | 12.3 | 8.1 | 8.0 |
| **Random *(BCL)*** | 56 | 26.5 | 65.3 | 30.8 | 56.5 | 36.3 | 76.5 |
| **Random (√N)** | 120 | 50.9 | 74.8 | 57.7 | 69.1 | 48.2 | 79.8 |

| *Ertugliflozin* | *Number of analogues* | *SpaceCov HRC_ward* | *PopCov HRC_ward* | *SpaceCov KMN* | *PopCov KMN* | *SpaceCov KMED* | *PopCov KMED* |
|---|---|---|---|---|---|---|---|
| **Bibliographic data *(BD)*** | 21 | 7.5 | 8.0 | 8.3 | 8.5 | - | - |
| **Random *(BD)*** | 21 | 15.9 | 18.6 | 15.9 | 17.9 | - | - |
| **Combinatorial Biblio data *(BCL)*** | 56 | 10.0 | 10.5 | 11.7 | 11.9 | - | - |
| **Random *(BCL)*** | 56 | 36.2 | 41.5 | 36.5 | 40.4 | - | - |
| **Random (√N)** | 120 | 60.2 | 66.6 | 61.1 | 65.9 | - | - |

**Table S12.** SC and PC for Rufinamide analogues ($k = \sqrt{N}$).

| *Rufinamide* | *Number of analogues* | *SpaceCov HRC_ave* | *PopCov HRC_ave* | *SpaceCov HRC_complete* | *PopCov HRC_ complete* | *SpaceCov OV_BIN* | *PopCov OV_BIN* |
|---|---|---|---|---|---|---|---|
| **Bibliographic data *(BD)*** | 22 | 11.6 | 2.0 | 7.4 | 1.1 | 8.4 | 2.2 |
| **Random *(BD)*** | 22 | 15.0 | 54.1 | 18.3 | 39.1 | 20.5 | 56.5 |
| **Combinatorial Biblio data *(BCL)*** | 144 | 28.4 | 8.3 | 23.2 | 8.3 | 31.0 | 9.7 |
| **Random *(BCL)*** | 144 | 46.0 | 86.6 | 56.1 | 84.8 | 54.0 | 90.4 |
| **Random (√N)** | 95 | 37.6 | 80.7 | 46.5 | 77.3 | 45.8 | 85.5 |

| *Rufinamide* | *Number of analogues* | *SpaceCov HRC_ward* | *PopCov HRC_ward* | *SpaceCov KMN* | *PopCov KMN* | *SpaceCov KMED* | *PopCov KMED* |
|---|---|---|---|---|---|---|---|
| **Bibliographic data *(BD)*** | 22 | 5.3 | 3.2 | 6.3 | 2.7 | 4.2 | 2.9 |
| **Random *(BD)*** | 22 | 20.3 | 24.5 | 20.3 | 24.7 | 20.3 | 25.0 |
| **Combinatorial Biblio data *(BCL)*** | 144 | 12.6 | 8.7 | 15.8 | 8.7 | 15.8 | 11.8 |
| **Random *(BCL)*** | 144 | 72.9 | 80.4 | 73.0 | 80.1 | 72.1 | 80.4 |
| **Random (√N)** | 95 | 59.6 | 67.5 | 59.4 | 67.3 | 59.1 | 67.7 |

**Table S13.** SC and PC for Azilsartan Medoxomil analogues ($k = \sqrt{N}$).

| *Azilsartan Medoxomil* | *Number of analogues* | *SpaceCov HRC_ave* | *PopCov HRC_ave* | *SpaceCov HRC_complete* | *PopCov HRC_ complete* | *SpaceCov OV_BIN* | *PopCov OV_BIN* |
|---|---|---|---|---|---|---|---|
| **Bibliographic data *(BD)*** | 4 | 11.8 | 0.5 | 8.8 | 0.5 | 13.0 | 0.5 |
| **Random *(BD)*** | 4 | 8.8 | 52.7 | 10.5 | 25.4 | 15.0 | 33.6 |
| **Combinatorial Biblio data *(BCL)*** | 9 | 26.5 | 1.9 | 20.6 | 5.6 | 26.1 | 3.9 |
| **Random *(BCL)*** | 9 | 14.5 | 69.3 | 20.3 | 45.4 | 27.1 | 57.6 |
| **Random (√N)** | 34 | 30.5 | 84.8 | 45.6 | 79.2 | 51.0 | 88.8 |

| *Azilsartan Medoxomil* | *Number of analogues* | *SpaceCov HRC_ward* | *PopCov HRC_ward* | *SpaceCov KMN* | *PopCov KMN* | *SpaceCov KMED* | *PopCov KMED* |
|---|---|---|---|---|---|---|---|
| **Bibliographic data *(BD)*** | 4 | 8.8 | 1.3 | 8.8 | 1.3 | 5.9 | 1.3 |
| **Random *(BD)*** | 4 | 11.0 | 15.7 | 11.1 | 14.5 | 11.1 | 13.5 |
| **Combinatorial Biblio data *(BCL)*** | 9 | 20.6 | 10.6 | 20.6 | 11.2 | 8.8 | 3.3 |
| **Random *(BCL)*** | 9 | 22.6 | 31.6 | 22.8 | 29.5 | 23.0 | 27.6 |
| **Random (√N)** | 34 | 56.1 | 72.6 | 57.8 | 71.4 | 60.3 | 68.4 |

**Table S14.** SC and PC for Leflunomide analogues ($k = \sqrt{N}$).

| *Leflunomide* | *Number of analogues* | *SpaceCov HRC_ave* | *PopCov HRC_ave* | *SpaceCov HRC_complete* | *PopCov HRC_ complete* | *SpaceCov OV_BIN* | *PopCov OV_BIN* |
|---|---|---|---|---|---|---|---|
| **Bibliographic data *(B)*** | 114 | 28.9 | 16.2 | 34.2 | 21.3 | 29.2 | 36.2 |
| **Random (*B*)** | 114 | 62.8 | 84.7 | 65.5 | 82.7 | 59.9 | 87.7 |
| **Combinatorial Biblio data *(C)*** | 2,844 | 76.3 | 67.5 | 84.2 | 77.3 | 70.8 | 86.2 |
| **Random *(C)*** | 2,844 | 99.0 | 100.0 | 99.6 | 100.0 | 98.0 | 99.9 |
| **Random (√N)** | 76 | 52.8 | 74.7 | 54.3 | 72.4 | 50.8 | 80.8 |

| *Leflunomide* | *Number of analogues* | *SpaceCov HRC_ward* | *PopCov HRC_ward* | *SpaceCov KMN* | *PopCov KMN* | *SpaceCov KMED* | *PopCov KMED* |
|---|---|---|---|---|---|---|---|
| **Bibliographic data *(B)*** | 114 | 22.4 | 16.4 | 22.4 | 17.9 | 22.4 | 23.3 |
| **Random (*B*)** | 114 | 74.2 | 79.8 | 74.7 | 79.7 | 74.1 | 79.9 |
| **Combinatorial Biblio data *(C)*** | 2,844 | 76.3 | 74.4 | 75.0 | 73.7 | 86.8 | 88.7 |
| **Random *(C)*** | 2,844 | 100.0 | 100.0 | 100.0 | 100.0 | 100.0 | 100.0 |
| **Random (√N)** | 76 | 60.7 | 66.8 | 61.1 | 66.3 | 60.7 | 66.8 |

**Table S15.** List of 206 1D and 2D descriptors calculated using MOE2020.09

| | | | |
|---|---|---|---|
| apol | chi0 | mr | Q_VSA_HYD |
| ast_fraglike | chi0v | mutagenic | Q_VSA_NEG |
| ast_fraglike_ext | chi0v_C | nmol | Q_VSA_PNEG |
| ast_violation | chi0_C | opr_brigid | Q_VSA_POL |
| ast_violation_ext | chi1 | opr_leadlike | Q_VSA_POS |
| a_acc | chi1v | opr_nring | Q_VSA_PPOS |
| a_acid | chi1v_C | opr_nrot | radius |
| a_aro | chi1_C | opr_violation | reactive |
| a_base | chiral | PC+ | rings |
| a_count | chiral_u | PC- | RPC+ |
| a_don | density | PEOE_PC+ | RPC- |
| a_donacc | diameter | PEOE_PC- | rsynth |
| a_heavy | FCharge | PEOE_RPC+ | SlogP |
| a_hyd | GCUT_PEOE_0 | PEOE_RPC- | SlogP_VSA0 |
| a_IC | GCUT_PEOE_1 | PEOE_VSA+0 | SlogP_VSA1 |
| a_ICM | GCUT_PEOE_2 | PEOE_VSA+1 | SlogP_VSA2 |
| a_nB | GCUT_PEOE_3 | PEOE_VSA+2 | SlogP_VSA3 |
| a_nBr | GCUT_SLOGP_0 | PEOE_VSA+3 | SlogP_VSA4 |
| a_nC | GCUT_SLOGP_1 | PEOE_VSA+4 | SlogP_VSA5 |
| a_nCl | GCUT_SLOGP_2 | PEOE_VSA+5 | SlogP_VSA6 |
| a_nF | GCUT_SLOGP_3 | PEOE_VSA+6 | SlogP_VSA7 |
| a_nH | GCUT_SMR_0 | PEOE_VSA-0 | SlogP_VSA8 |
| a_nI | GCUT_SMR_1 | PEOE_VSA-1 | SlogP_VSA9 |
| a_nN | GCUT_SMR_2 | PEOE_VSA-2 | SMR |
| a_nO | GCUT_SMR_3 | PEOE_VSA-3 | SMR_VSA0 |
| a_nP | h_ema | PEOE_VSA-4 | SMR_VSA1 |
| a_nS | h_emd | PEOE_VSA-5 | SMR_VSA2 |
| balabanJ | h_emd_C | PEOE_VSA-6 | SMR_VSA3 |
| BCUT_PEOE_0 | h_logD | PEOE_VSA_FHYD | SMR_VSA4 |
| BCUT_PEOE_1 | h_logP | PEOE_VSA_FNEG | SMR_VSA5 |
| BCUT_PEOE_2 | h_logS | PEOE_VSA_FPNEG | SMR_VSA6 |
| BCUT_PEOE_3 | h_log_dbo | PEOE_VSA_FPOL | SMR_VSA7 |
| BCUT_SLOGP_0 | h_log_pbo | PEOE_VSA_FPOS | TPSA |
| BCUT_SLOGP_1 | h_mr | PEOE_VSA_FPPOS | VAdjEq |
| BCUT_SLOGP_2 | h_pavgQ | PEOE_VSA_HYD | VAdjMa |
| BCUT_SLOGP_3 | h_pKa | PEOE_VSA_NEG | VDistEq |
| BCUT_SMR_0 | h_pKb | PEOE_VSA_PNEG | VDistMa |
| BCUT_SMR_1 | h_pstates | PEOE_VSA_POL | vdw_area |
| BCUT_SMR_2 | h_pstrain | PEOE_VSA_POS | vdw_vol |
| BCUT_SMR_3 | Kier1 | PEOE_VSA_PPOS | vsa_acc |
| bpol | Kier2 | petitjean | vsa_acid |
| b_1rotN | Kier3 | petitjeanSC | vsa_base |
| b_1rotR | KierA1 | Q_PC+ | vsa_don |
| b_ar | KierA2 | Q_PC- | vsa_hyd |
| b_count | KierA3 | Q_RPC+ | vsa_other |
| b_double | KierFlex | Q_RPC- | vsa_pol |
| b_heavy | lip_acc | Q_VSA_FHYD | Weight |
| b_max1len | lip_don | Q_VSA_FNEG | weinerPath |
| b_rotN | lip_druglike | Q_VSA_FPNEG | weinerPol |
| b_rotR | lip_violation | Q_VSA_FPOL | zagreb |
| b_single | logP(o/w) | Q_VSA_FPOS | |
| b_triple | logS | Q_VSA_FPPOS | |
